# Continuous spark plasma synthesis of Au/Co binary nanoparticles with tunable properties

**DOI:** 10.1038/s41598-022-22928-0

**Published:** 2022-11-03

**Authors:** Lajos Péter Villy, Attila Kohut, Albert Kéri, Ádám Bélteki, György Radnóczi, Zsolt Fogarassy, György Zoltán Radnóczi, Gábor Galbács, Zsolt Geretovszky

**Affiliations:** 1 Department of Optics and Quantum Electronics, University of Szeged, Dóm Sq. 9, 6720 Szeged, Hungary; 2 Department of Materials Science, Interdisciplinary Excellence Centre, University of Szeged, Dugonics Sq. 13, 6720 Szeged, Hungary; 3 Department of Inorganic and Analytical Chemistry, University of Szeged, Dóm Sq. 7, 6720 Szeged, Hungary; 4 Centre for Energy Research, Konkoly-Thege St. 29-33, 1121 Budapest, Hungary

**Keywords:** Nanoscale materials, Synthesis and processing

## Abstract

We present here a scalable and environmentally friendly gas phase technique employing atmospheric pressure electrical spark discharge plasmas for the production of Au/Co binaries, an effective catalyst system for the decomposition of hydrogen-rich compounds, such as ammonium borane. We demonstrate that Au/Co alloy nanoparticles can be produced via the spark plasma-based technique. The possibility of varying the morphology and phase structure via real time heat treatment of the generated aerosol to form Au/Co/CoO particles with continuous control over a wide particle compositional range (from 24 to 64 at.% [Co]/([Co] + [Au]) content) is also demonstrated. Since our spark-based approach is proven to be capable of providing reasonable particle yields, these results may contribute to the transition of lab-scale, nanocatalyst-based hydrogen storage systems to real world applications.

## Introduction

Binary nanoparticles (BNPs) attract considerable interest due to their improved magnetic, optical or catalytic performance in many fields, both in alloy^1,2^ and phase segregated forms^3,4^. In case of catalysis, a prominent example is the Au/Co system, which is a suitable candidate for generating hydrogen from ammonia borane (amminetrihydridoboron), a promising condensed-phase fuel material for hydrogen-powered engines^5–9^. Au/Co binary NPs (BNPs) are usually synthesized via chemical methods, e.g., by the simultaneous reduction of Au and Co precursors^8,10–12^ or by employing cobalt^13,14^ or gold^15^ NPs as seeds. These techniques may result in Au@Co^8,15^ or Co@Au^13^ core–shell BNPs as well as Au/Co nanoalloys^11,12^ depending on the experimental conditions. Chemical methods inherently use various solvents and reagents in usually fairly complex, multi-step processes, where the size- and composition-control of the synthesized Au/Co BNPs are challenging, which is a limiting factor for studying their applications. Much better control of the Au/Co BNPs can be obtained via gas-phase methods, where particle formation is facilitated by the condensation and aggregation of metal atoms and ions in a gaseous or evacuated environment^16,17^. Mayoral et al. have shown that both Au@Co and Co@Au core–shell BNPs with controlled size and composition can be generated by the inert gas condensation method, utilizing a supersaturated metal vapor formed by sputtering of a bulk target^17,18^. Llamosa et al. have shown that tailoring of Au/Co BNPs can be achieved by using multiple ion cluster sources to produce well-defined core–shell, or core–shell-shell structures in ultra-high vacuum^16^. These approaches offer extensive control over the particle formation process, however, they require sophisticated instrumentation and/or a high vacuum environment, which makes their scalability to industrial level and hence their real-life application highly challenging. Another physical, gas-phase method, which has great potential both in versatility and scalability is spark ablation^19^. It is based on a technically simple idea, namely, the erosion of two conducting electrodes by high-voltage and high-current, microsecond-long, oscillatory, repetitive sparking^20^. Similarly to the gas-phase techniques mentioned above, the process only includes high-purity bulk electrodes and a controlled, gaseous environment, hence exceptionally pure NPs can be obtained^21^. In addition, spark ablation does not require a vacuum system and its electrical implementation is also simple, which facilitates scalable particle generation^22,23^. Moreover, due to the ability of periodically eroding two bulk electrodes with different compositions, spark ablation has utmost potential in the field of multielement NP synthesis with controllable composition and structure^24–27^. In the present paper we report on the spark ablation-based synthesis and characterization of Au/Co BNPs. We also demonstrate the possibility of tuning the composition of the Au/Co BNPs over a broad range, along with the variation of particle morphology. We believe that our results facilitate the development of methodologies to produce Au/Co nanocatalysts suitable both in quality and quantity for various real-life challenges, such as those related to hydrogen storage systems.

## Experimental

### Particle generation

**Figure 1:**
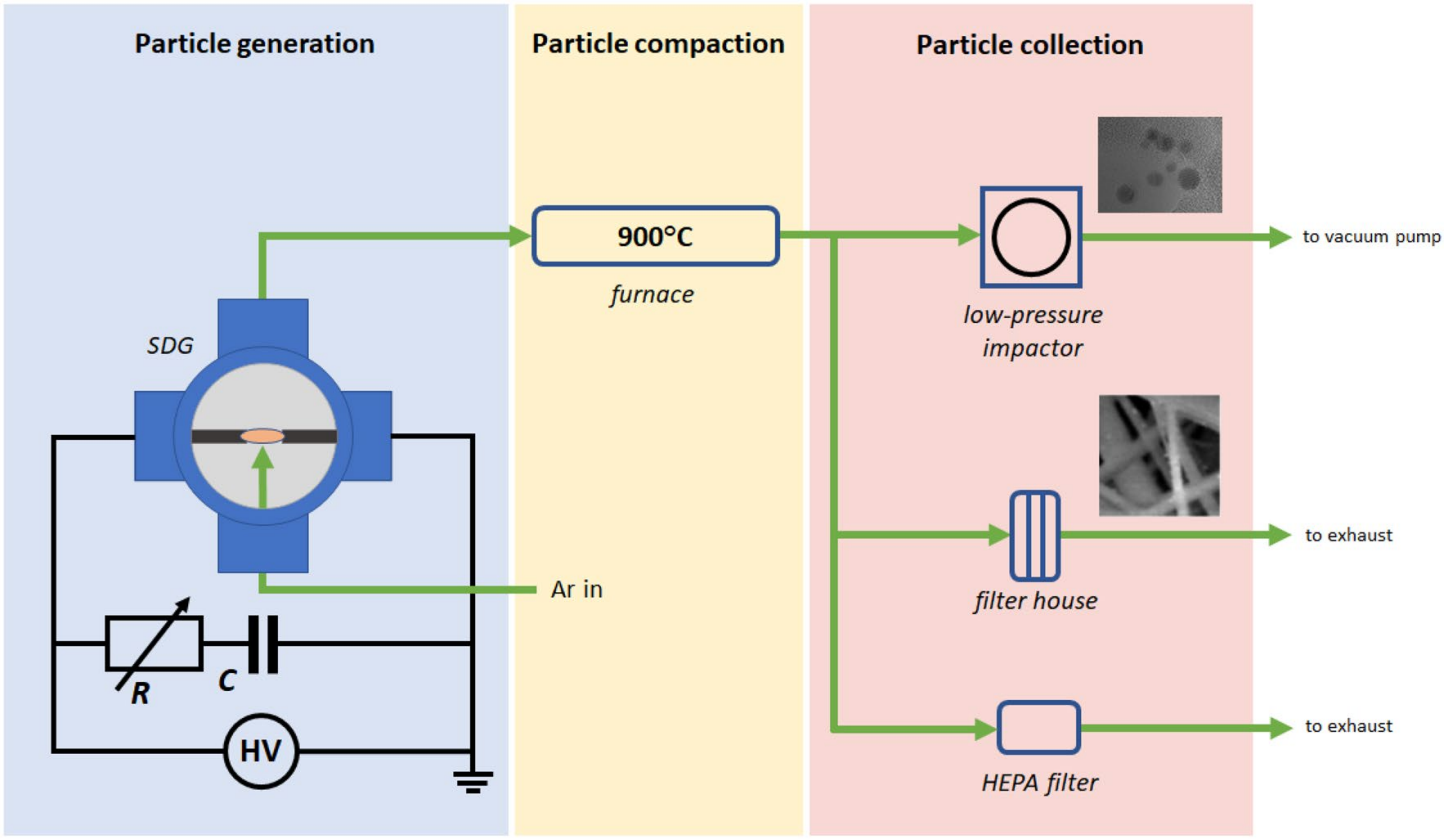
Schematic figure of the particle generation, collection, and sampling setup.

The experimental setup used in the present study is schematically shown in Fig. 1. The main part of the system is the spark discharge generator (SDG), a leak tight, stainless steel cylindrical chamber of DN-160-size, with four radially oriented, KF-40-size ports (Pfeiffer Vacuum GmbH). The chamber was oriented in an upright position, i.e. with the two large KF-160 ports facing to sides. 3.0 mm diameter Co (99.9% purity, Goodfellow Cambridge Ltd.) and Au (99.99% purity, Kurt J. Lesker Co.), cylindrical electrodes were used in our measurements, which were horizontally positioned and axially aligned. The gap between the two electrodes was 2.0 mm for all experiments. It was controlled by micropositioners (Model K150-BLM-1, MDC Vacuum Ltd.).

The argon (99.996% purity, Messer Hungarogáz Kft.) carrier gas flow was fed in the chamber via one of the KF-40 ports (upward pointing “crossflow”) through a 2.85 mm inner diameter injector nozzle, with its tip placed at the midpoint between the electrodes at a distance of 4.25 mm from the common axis of the two electrodes. The generated particles left the chamber via a 4.7 mm inner diameter outlet port at the top of the chamber, 153.3 mm from the electrodes’ axis. The gas flow rate was controlled by a mass flow controller (Model GFC16, Aalborg Instruments & Controls, Inc.) and set to 5 standard liters per minute. The spark chamber was evacuated—reaching a pressure of ca. 90 mbar—prior to sparking by means of a diaphragm pump then refilled with argon, which was kept flowing through the system during all experiments. Particle generation was carried out slightly above atmospheric pressure and monitored by a pressure gauge (Model VD81, Thyracont Vacuum Instruments GmbH).

Spark discharges were generated by a capacitor charging circuit. A monolithic, high voltage capacitor (Model 450PM980, General Atomics) of 8 nF capacitance was continuously charged by a capacitor charging power supply (Model HCK 800–12,500, FuG GmbH). Spark discharges were created between the Au and Co electrodes when the capacitor’s voltage exceeded the breakdown voltage of the electrode gap, mainly affected by the materials of the electrodes and the carrier gas, the gap distance, and the temperature in the vicinity of the electrodes. The resulting spark discharge is a bipolar, dampened, oscillatory discharge. The spark repetition rate was controlled by the charging current of the capacitor and kept constant at 100 Hz. The total resistance of the discharge loop was varied in the range of 1–9 Ω by using FeCrAl alloy wires (Kanthal, Sandvik Intellectual Property AB, Sweden) of different length. The voltage and current waveforms in the discharge circuit were measured by a broad-band high voltage probe (Model P6015A, Tektronix Inc.) and a current probe (Model 110, Pearson Electronics, Inc.), and visualized and recorded on a digital oscilloscope (Model DSOX2024A, Keysight Technologies Inc.).

### Particle characterization

The particle characterization used in the present experiments is only summarized briefly below, since it is described in more detail elsewhere^28^. The created NPs were collected on glass microfiber filters (GF/A CAT No. 1820–047, Whatman plc, part of GE Healthcare Life Sciences, General Electric), placed in a filter holder (Advantec AS). The sample collection time was 30 min. Particles were generated both with- and without heat treatment. For in-line heat treatment of the NPs the generated aerosol was passed through a 900 °C tube furnace. According to our CFD simulations, the residence time of the particles was 12 s in the tube. However, the portion of the tube in which the temperature is around 900 °C is only about 20 cm long, so the aerosol particles spend about 3.2 s at 900 °C. After each particle sampling, the tubing and the filter holders were cleaned in an ultrasonic bath (Ultrasonic 300, NEY, now Blackstone-NEY Ultrasonics) using a solution which contained 50% ethanol (96% purity, Molar Chemical Ltd.) and 50% trace-quality de-ionized water. Samples were stored in closed Petri dishes until composition analysis. An inductively coupled plasma mass spectrometer (ICP-MS, 7700x, Agilent Technologies Inc.) was used for the determination of the elemental composition of the heat-treated NPs. Sample dissolution was carried out by aqua regia, prepared freshly from trace quality cc. hydrochloric and cc. nitric acids (VWR International, LLC.) under 16 h of contact time. The resulting clear solutions were filtered through 0.22 μm PTFE membrane filters and diluted with trace-quality de-ionized labwater (MilliPore Elix 10 equipped with a Synergy polishing unit, Merck GmbH.) prior to analysis. Multipoint, matrix-matched calibration was performed using certified calibration standards (IV-ICPMS-71A and IV-ICPMS-71C, Inorganic Ventures). ICP-MS plasma and interface parameters were optimized using standard tuning solutions (G1820-60,410, Agilent). All ICP-MS measurements were carried out by monitoring the signal of the 59Co and 197Au isotopes, in He mode, using the ORS3 collision cell. Data processing was performed within the Agilent Mass Hunter software. The 99.996% purity argon and 99.999% purity helium gases were provided by Messer Hungarogáz Kft.

For morphological characterization the generated particles were sampled onto lacey carbon copper grids (S166 Lacey Carbon Film 200 Mesh Cu, Agar Scientific Ltd.) by using a low-pressure impactor. The morphology of the nanoparticles was analyzed by high-resolution transmission electron microscopy (HRTEM) using a Philips CM20 at 200 kV, and a FEI Titan-Themis (scanning) transmission electron microscope ((S)TEM) with Cs-corrected objective lens, in both HREM and STEM modes (also at 200 kV, point resolution being around 0.08 nm in HREM mode and 0.16 nm in STEM mode). EDS analysis was performed to obtain compositional maps of the samples (Themis Super-X EDS detector) in STEM mode. For EDS quantification the background was determined using a parabolic multi-polynomial model choosing the background windows automatically by the software (Velox 2.10) and then checked visually on the spectrum if any manual corrections were needed. For quantification of the peaks, the Brown-Powell ionization cross section model was applied.

Data processing was performed within the OriginPro (OriginPro 8.6 32bit, OriginLab Corporation, https://www.originlab.com) software and the Fiji (version: Fiji 2.9.0, https://imagej.net/software/fiji/) open-source platform for image analysis^28^.

## Results and discussion

### Morphology of the generated nanostructures

**Figure 2:**
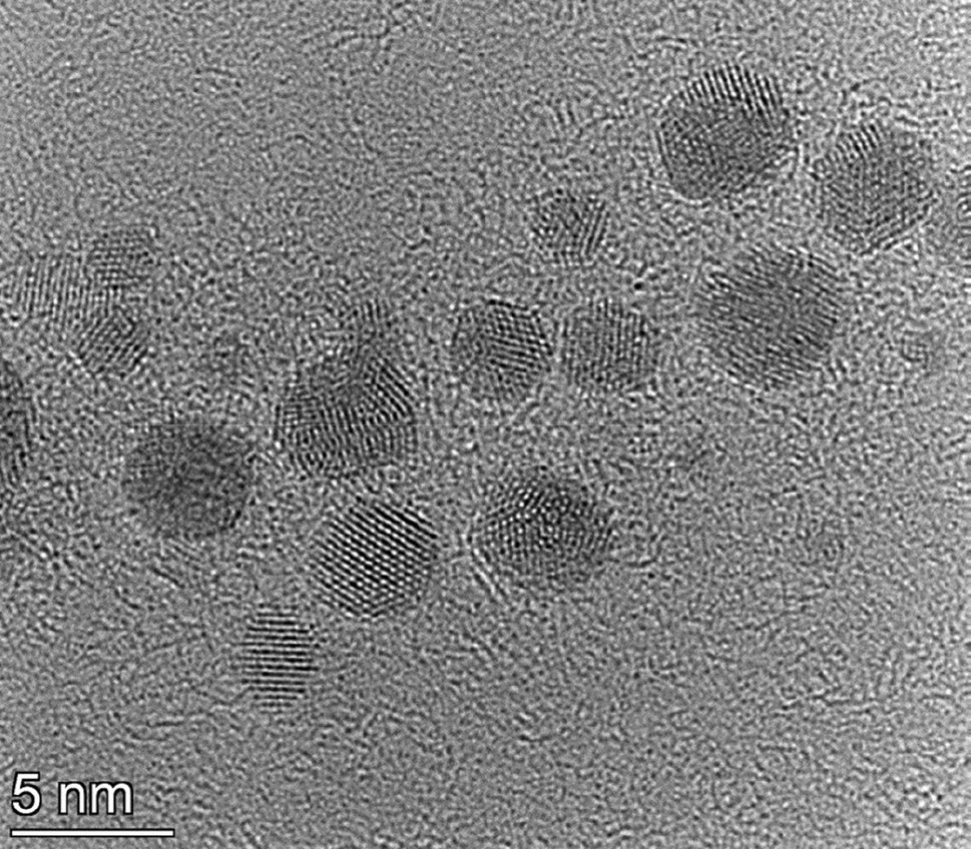
TEM micrograph of the generated Au/Co BNPs generated at 1.0 Ω circuit resistance.

BNP generation from dissimilar electrodes by spark ablation is facilitated by the oscillatory nature of the spark discharge initiated between the electrodes. Due to polarity reversals, both electrodes are ablated, and their material is released into the gas phase^21,29,30^. When dissimilar electrodes are used—e.g., gold and cobalt—this process makes the formation of BNPs from gold and cobalt atoms possible. Typical NPs produced in our experiments are shown in Fig. 2. It can be seen that primary particles form agglomerates, as expected under the experimental conditions used. Two morphologies are observable in the TEM micrographs: *i)* the mostly spherical (sometimes slightly elongated) crystalline particles are embedded in an *ii)* amorphous matrix, forming a more or less continuous structure around the higher contrast particles. Area-averaged EDS analysis of typical agglomerates—like that shown in Fig. 2—revealed that they are made up of both gold and cobalt atoms and have a typical Co content of ca. 35 at.%.

**Figure 3:**
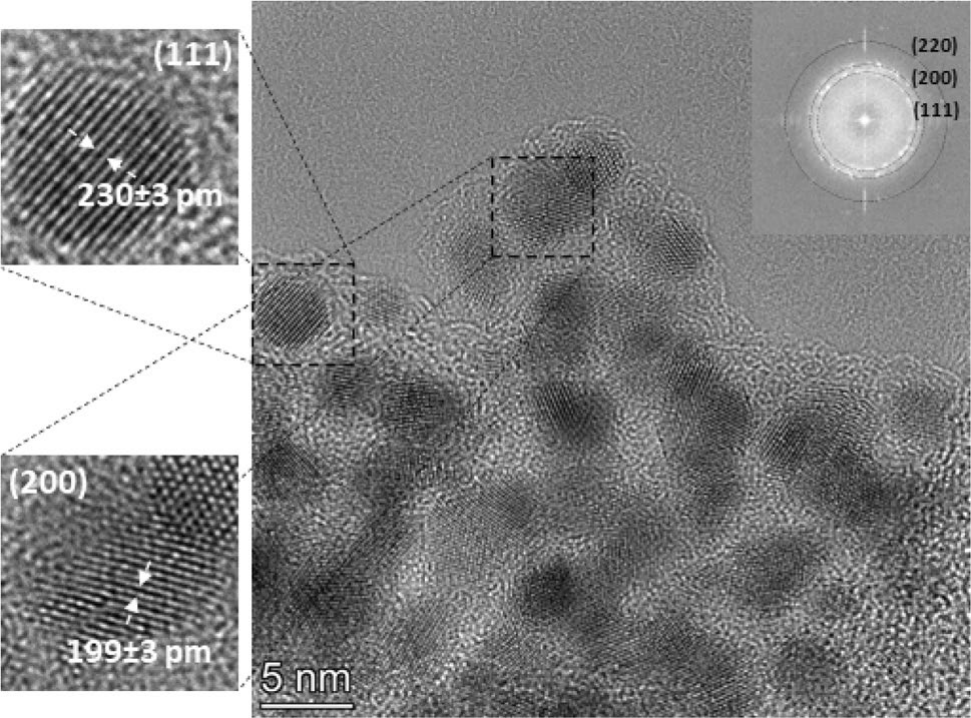
TEM micrograph of a typical aggregate of Au/Co BNPs (generated at 1.0 Ω circuit resistance), with close-ups of two different crystalline particles (on the left of the main image), showing the value of average lattice spacing obtained from the given region. The Fourier transform of the TEM micrograph of the whole aggregate is shown in the upper right corner of the image.

The analysis of high-resolution TEM images reveals that a crystalline phase is present in the sample, corresponding to a face-centered cubic (FCC) lattice structure (please see the Fourier transform (FT) of the TEM micrograph in the inset of Fig. 3). By measuring the lattice spacing in particles with different orientation, a lattice constant of about 398 pm was found. This is between the 407.8 pm and 354.3 pm values corresponding to pure gold and FCC cobalt, respectively. According to the tabulated data of Okamoto et al*.*, the lattice constant measured within our crystalline particles belongs to the Co content of about 25 at.%^31^. Considering that lattice constant of metals slightly differs in bulk and nano forms, the Co content of the crystalline particles is somewhat smaller than 25 at%. Since our EDS results indicate an area-averaged Co content within an aggregate (exemplified in Fig. 2) that is higher than the predicted Co content of the particles, the amorphous structure surrounding the crystalline particles should be cobalt-rich, and must contain more than 35 at% Co. This suggests that crystalline, gold-rich Au/Co alloy BNPs are generated, that are embedded in amorphous cobalt oxide (CoO_x_) matrix. The exclusion of amorphous Co is supported by two facts: 1) pure metals usually do not form amorphous phase and 2) EDS mapping revealed the correlation of the distribution of atomic cobalt and oxygen, as will be shown later in Fig. 8. The geometric mean size of the Au/Co BNPs is 4.50 ± 0.13 nm, as obtained from analysis of 227 particles from several TEM images. Numerical studies show that in this size range the Au–Co system tends to form core–shell equilibrium structures, with a preference towards an Au shell^3,32^. Experimental studies performed by using electron-beam deposition and a heated substrate also reported such phase segregation when the equilibrium structure is approached^33^. In case of spark ablation, however, mixing of different atoms of the two electrodes is associated with a highly transient process, namely the formation of the spark plasma. This is characterized by the fast cooling of the metal vapor (often referred to as quenching), which was identified as the main reason of the so-called kinetical trapping of alloy structures, even in case of material combinations which are immiscible in bulk^24^. This explains the alloy formation in our experiments, despite the tendency of Au and Co to form a core–shell structure in equilibrium. It should also be added that materials systems—such as Ag–Cu—with a bulk phase diagram closely resembling that of Au–Co also tend to form alloys at room temperature in the sub 5 nm range, until coalescence of the particles sets in^34^. Since coalescence of highly active small particles into larger ones is especially undesirable in catalysis, several strategies are employed to mitigate this process. One of them is the formation of protective oxide layers^35^. As evidenced by Fig. 2, in the present case, the formation of such protective layer is inherently realized, facilitated by the trace amount of oxygen in the spark chamber. Nevertheless, if a particular application requires purely metallic particles, oxidation can virtually be eliminated by adding H_2_ to the carrier gas of the spark discharge nanoparticle generator, as demonstrated in Refs.^24,36^.

### Heat treatment of the Au–Co aerosol

**Figure 4:**
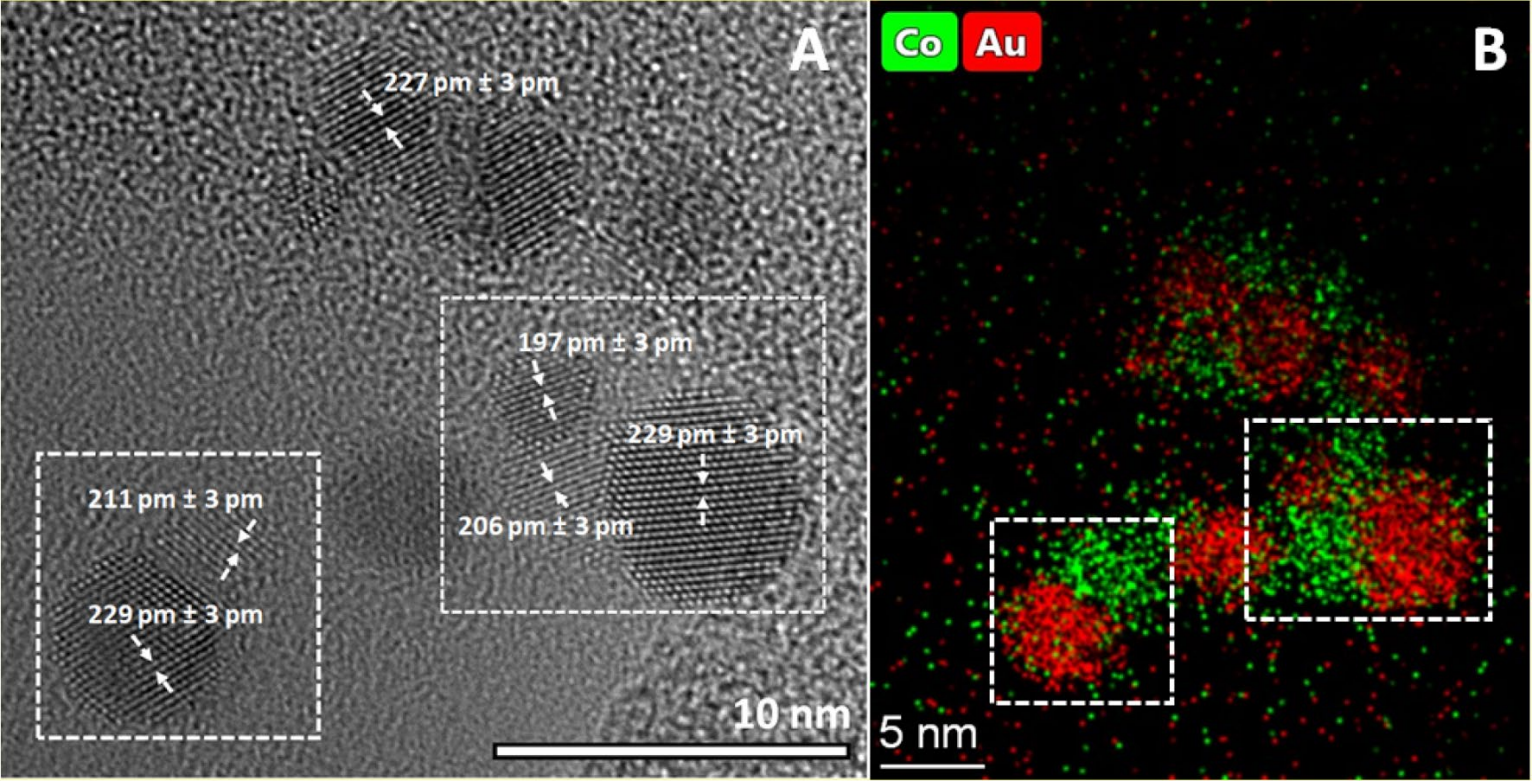
TEM image of Au/Co particles (generated at 1.0 Ω circuit resistance) after heat treatment at 900 °C (**A**), and elemental map of the same area as obtained by SEM–EDS analysis (**B**). Dashed squares indicate typical particles subjected to analysis.

As evidenced by the above results, spark-produced crystalline Au/Co nanoparticles are embedded in an amorphous CoO_x_ matrix. In order to study the possibility of varying the binary structure, the as formed Au–Co aerosol was exposed to heat treatment in a tube furnace (at 900 °C) after leaving the generator chamber. The resulting NPs can be seen in Fig. 4. It is apparent that smaller primaries are compacted into larger particles consisting of both Co and Au (cf. Figure 4). As evidenced by the STEM and EDS analysis, amorphous cobalt-oxide no longer covers individual Au/Co particles, instead, segregation is promoted and structures resembling “nano snowmen” are formed, consisting of spherical parts touching each other (see the squared areas in Fig. 4). It can be readily seen from the lattice planes observable in the TEM micrographs that both regions of each particle are crystalline, the abundance of amorphous structure is rather low (cf. Figure 4A), especially as compared to the non-heat-treated case. By measuring the lattice spacing in the two main regions—i.e., in those which appear to be gold- and cobalt-rich in the elemental map (Fig. 4B)—slightly lower lattice constants were obtained in the gold-rich areas than without heat treatment, indicating slightly higher cobalt content of the Au/Co particles. The cobalt-rich crystalline areas are found to have consistently higher lattice constant than that of pure cobalt, that is an evidence supporting the formation of crystalline CoO. According to the lattice constant analysis on high resolution TEM images and EDS-line scan data, cobalt(II) oxide is formed.

The above results well exemplify that the gas-phase nature of the process facilitates the practically real-time and continuous heat treatment of the produced particles, thereby offering additional control of particle properties, namely over their crystallinity and morphology. In the present case, heat treatment of the Au–Co aerosol in a tube furnace resulted in crystallization of the amorphous CoO_x_ and hence the formation of phase-segregated Au/Co/CoO particles.

### Composition-tuning of the heat-treated particles

**Figure 5:**
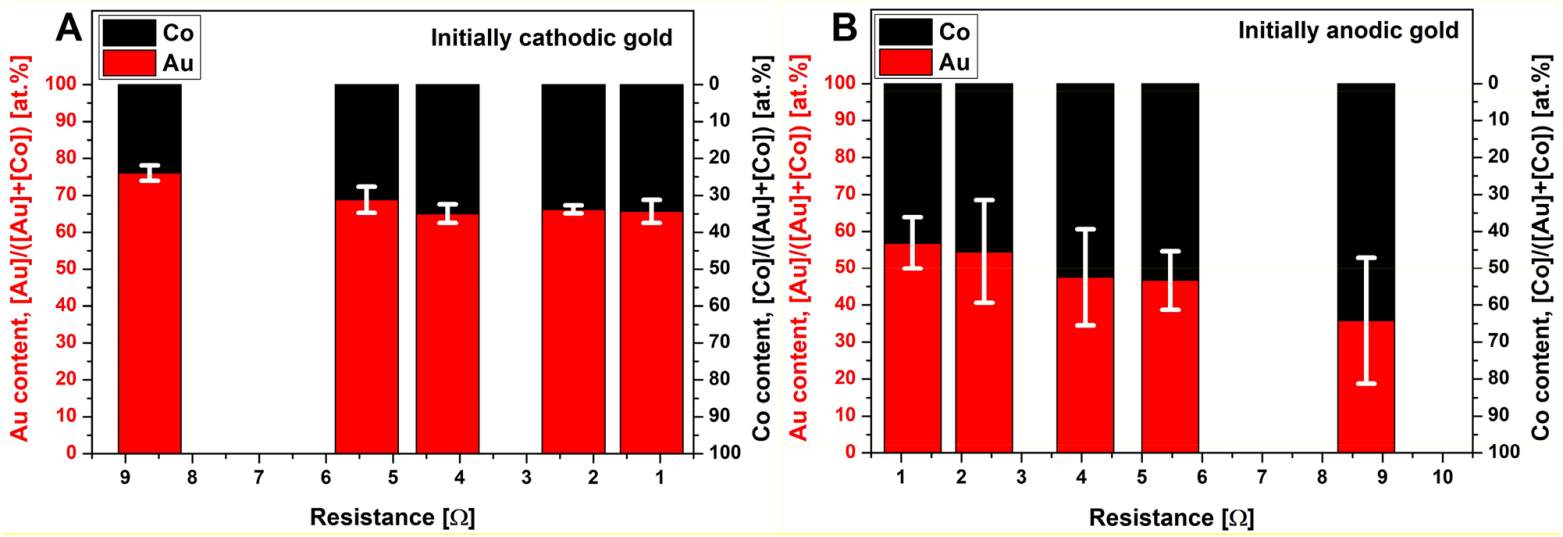
Variation of the composition of the Au/Co/CoO NPs obtained by ICP-MS, as a function of the total resistance of the discharge circuit when gold is initially cathodic (**A**) and anodic (**B**). Error bars indicate the uncertainty of the composition corresponding to a 90% confidence level.

The composition of the NPs generated by spark ablation depends on several factors, most notably on the properties of the electrode material, the initial polarity of the electrodes, and the electrical characteristics of the discharge circuit. This was recently formalized by both Feng et al.^30^ and Kohut et al.^25^. Their semiempirical spark mixing models correlate the energy deposited onto the two electrodes to their relative erosion and hence the average composition of the particles formed. Although some considerations of the two models differ, there is a consensus that the most influential parameter, affecting the relative erosion of the electrodes—and hence particle composition—is the shape of the current waveform measured in the discharge circuit^25,30^. More closely, composition is dominantly determined by the asymmetry of the waveform, defined as the ratio of the integrals corresponding to the negative and positive parts of the current waveform^25^. This ratio can effectively be tuned by the initial polarity of the electrodes and varying the total resistance of the discharge circuit^21,25,30^. We have previously shown the virtue of this approach for generating Au/Ag NPs with varying composition^25^. Here, we followed the same procedure with the intention to tune the average gold and cobalt content of the Au/Co/CoO NPs. The results—obtained by means of ICP-MS analysis of the generated BNPs, thus representing an average elemental composition of the entire product—are shown in Fig. 5. The tuning range achieved, expressed as [Co]/([Au] + [Co]) ratio of the NPs is ca. 24 to 64 at.%.

**Figure 6:**
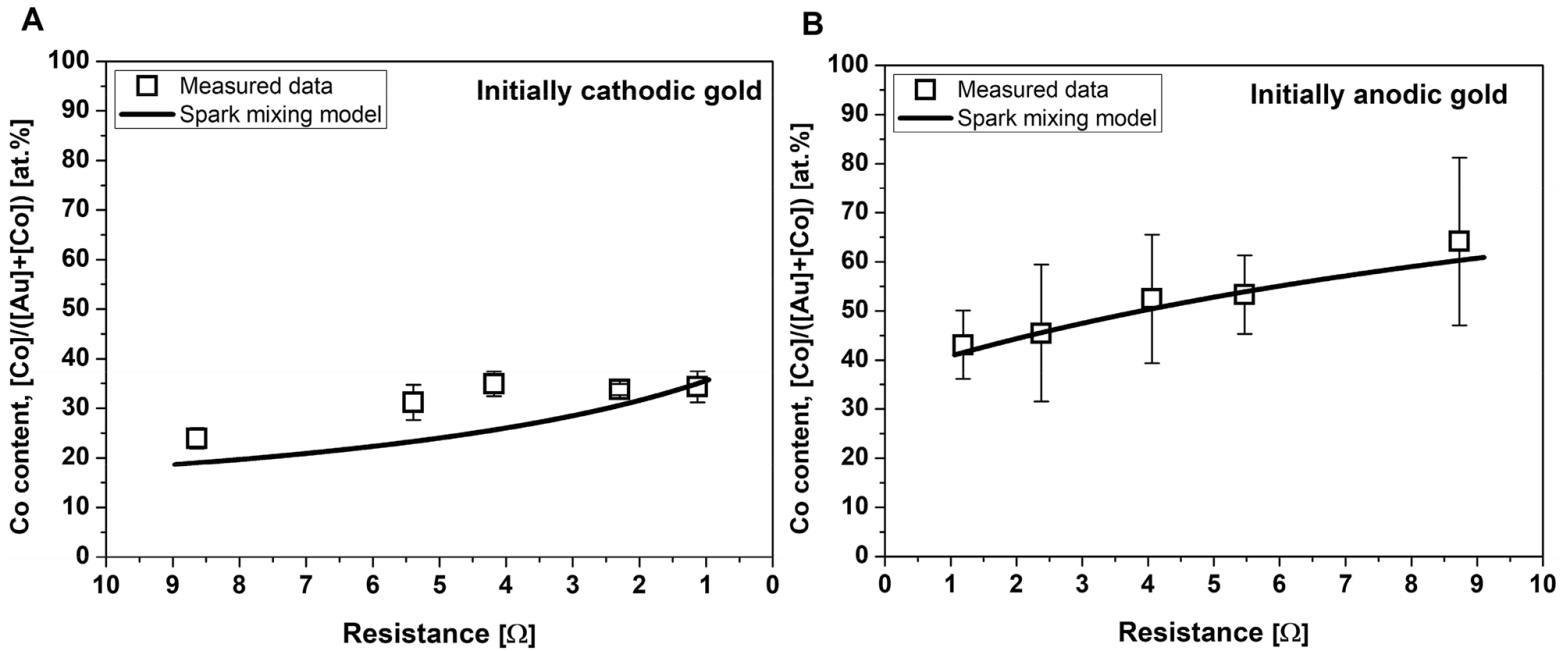
Comparison of the calculated (by using Eq. 1) and actual Co content of the generated Au/Co/CoO NPs with initially cathodic (**A**) and anodic (**B**) gold electrodes.

The experimentally obtained composition range was compared to the values calculated from our semiempirical spark mixing model^25^. This results in the following formula that describes the average atomic percentage of the material of the initially cathode electrode material in the generated NPs with respect to the total amount originating from both electrodes (${\varphi }_{C})$:

1 $${\varphi }_{C}=\frac{1}{\frac{1}{\frac{{C}_{A}}{{C}_{C}}\cdot \frac{{U}_{-}^{C}}{{U}_{-}^{A}}\cdot \frac{{k}^{{{\prime}}}+{U}_{+/-}^{C}}{{U}_{+/-}^{A}\cdot {k}^{{{\prime}}}+1}}+1},$$

where ${U}_{-}^{C}$ and ${U}_{-}^{A}$ are the cathode fall voltage of the initially cathodic and anodic electrode, respectively, and ${U}_{+/-}^{C}$ and ${U}_{+/-}^{A}$ are the ratio of the anode and cathode fall voltages for the initial cathode and anode electrodes. In Eq. (1), *k’* is the ratio of the temporal integrals of positive and negative currents whereas *C*_*A*_ and *C*_*C*_ are proportionality factors for the anode and cathode, which can be calculated from the thermal properties of the electrode materials^25,30^. For the calculation of the Co content of the Au/Co/CoO NPs by Eq. (1), one needs to know the cathode and anode fall voltages ${U}_{-}^{C}$ and ${U}_{-}^{A}$ of the two electrode materials. For cathodic gold, we have obtained ca. ${U}_{-}^{C}$= 60 V fall voltage in a separate study, along with the observation of a negligible anodic erosion^25^. However, we are unaware of any similar value for cobalt, but it can be estimated using the particle mass data determined by ICP-MS analysis and fitting the model to the measured values (the details of this numerical approach are described in^25^). We obtained ${U}_{-}^{C}=$ 28 V for the cathode fall voltage of cobalt. Since only the measured mass data corresponding to one of the two possible initial polarities was used to obtain the required constant for Co, the results can be cross checked by comparing the modeling results with the experiments performed at switched polarities, as shown in Fig. 6. It can be seen that the model (calculated by using Eq. 1) predictions are reasonably close to the experimental results at both initial polarities; the maximum relative deviation from the measured values is only 9%.

### Morphology of the Au/Co primary particles at increased total resistance

**Figure 7:**
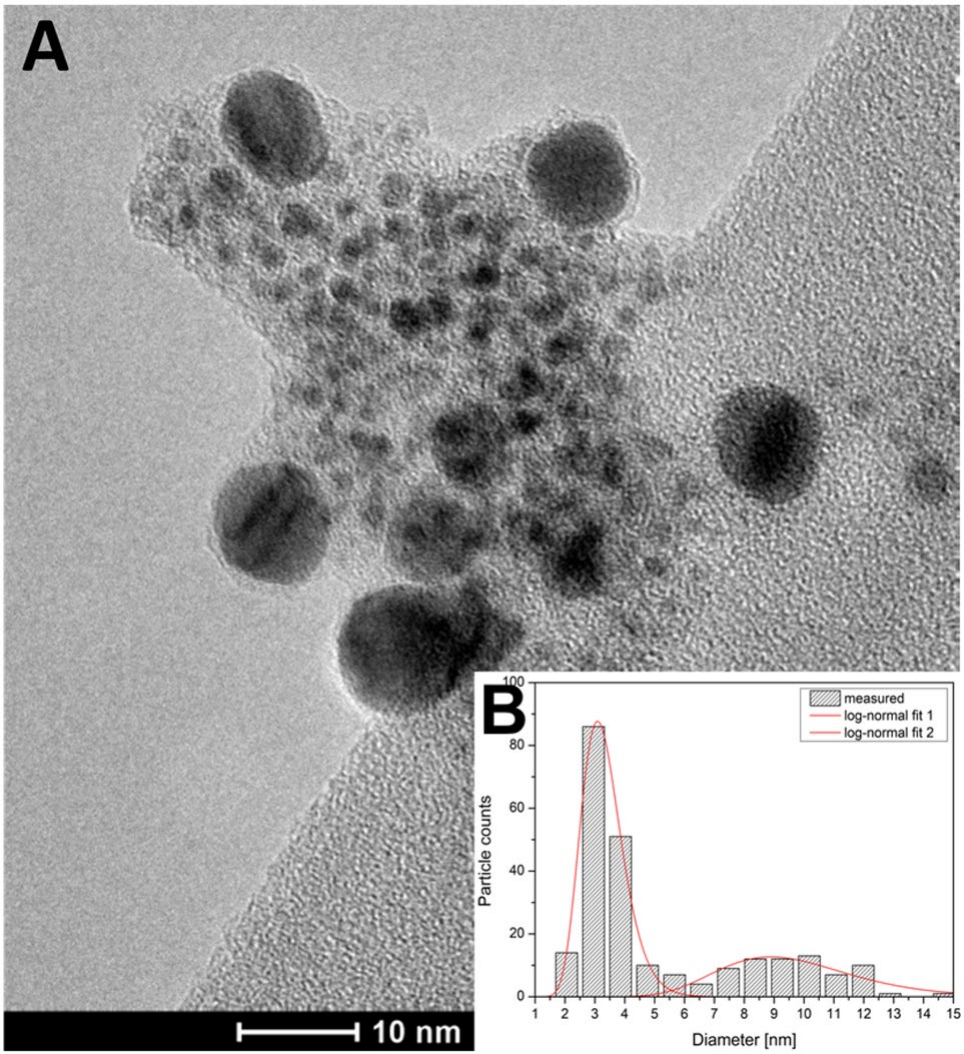
TEM micrograph of Au/Co BNPs generated at 5.4 Ω circuit resistance without heat-treatment (**A**), and the size distribution of 237 particles (**B**).

The variation of the total resistance not only affects the asymmetry of the current waveform, but the peak current and hence the spark energy, as well^37^, which in turn affects the primary particle size. Since the increase of total resistance results in decreasing spark energy, a decreasing primary size is expected^38^. This qualitative trend is well reflected in the TEM micrograph of particles generated at 5.4 Ω total resistance without heat treatment, shown in Fig. 7A. By deriving the size distribution of the Au/Co particles, a modal diameter of ca. 3 nm is obtained (see Fig. 7B). However, the size distribution exhibits a second peak with a mode around 9 nm, reflecting the presence of larger particles. The number concentration of these larger particles is lower than that of the smaller ones, and they exhibit a fairly spherical shape as evidenced by the TEM micrograph shown in Fig. 7A.

**Figure 8:**
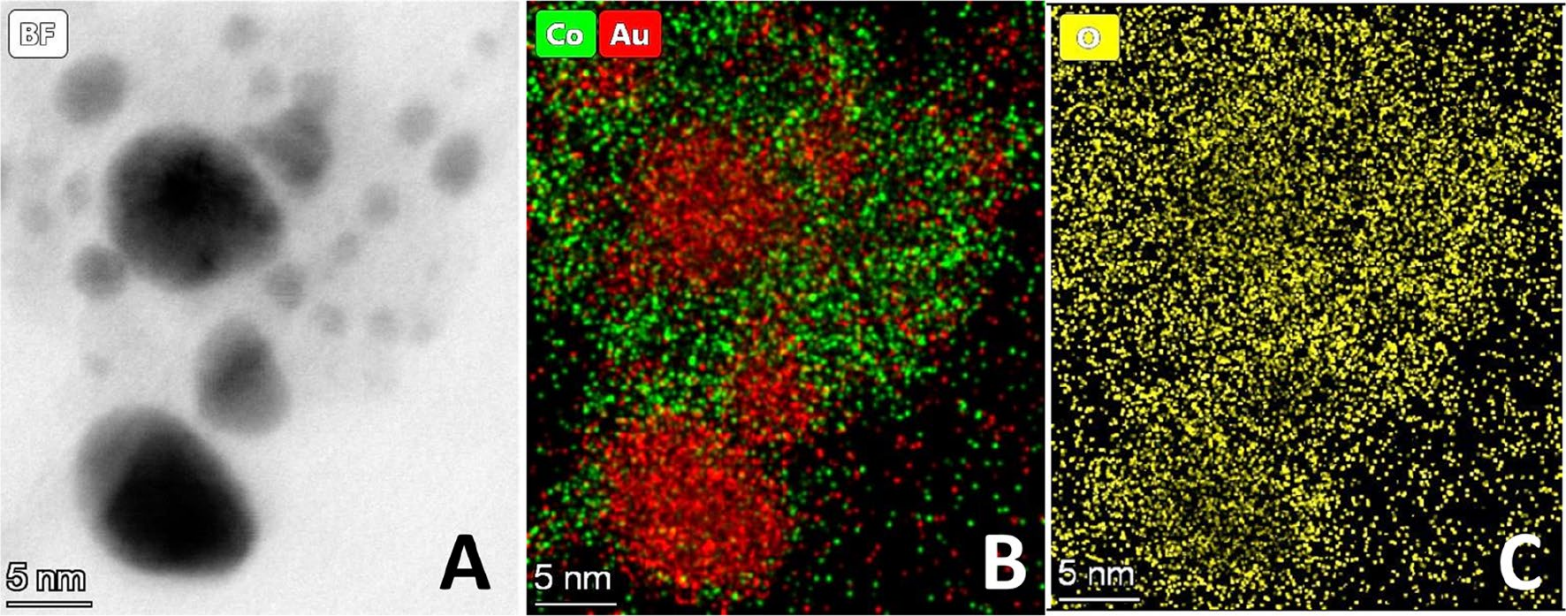
Bright field STEM image of Au/Co particles (generated at 5.4 Ω circuit resistance without heat treatment) (**A**), and SEM–EDS elemental maps of the same area showing the distribution of Co and Au (**B**), and O (**C**), respectively.

Similarly to that of the low-resistance case shown above, the crystalline particles are embedded in a continuous amorphous matrix, thus exhibiting two distinctly different structural features. To gain more insight into the effect of increased total resistance on the structure of the generated BNPs, STEM measurements have been performed along with EDS elemental mapping of Co, Au and O, as summarized in Fig. 8. Figure 8A shows a typical area of the sample where both of the two morphologies are present. As expected, the higher contrast areas are rich in gold, while the surrounding, brighter area is abundant in cobalt, clearly shown in the combined Au/Co elemental map in Fig. 8B. It can also be seen in Fig. 8C that the distribution of the oxygen is inhomogeneous and its higher abundance correlates to the cobalt-rich regions, which is in qualitative agreement with the presence of CoO_x_ in the amorphous regions. Thus, it can be stated that, unlike heat-treatment, increasing the resistance of the SDG does not change the overall crystal structure of the produced particles.

## Conclusions

In the present study the gas phase synthesis of Au/Co binary nanoparticles was demonstrated in atmospheric pressure electrical spark discharge plasmas. We have shown that the technique—when performed without additional heat treatment—essentially results in gold-rich crystalline Au/Co particles, embedded in an amorphous CoO_x_ matrix. It was shown that the morphology of the Au/Co binaries can effectively be changed by heat treatment of the as produced nano aerosol, that promotes the formation of polycrystalline Au/Co particles. This approach was used to demonstrate the generation of Au/Co/CoO “nanosnowmen” structure, consisting of gold-rich and cobalt-rich units of nearly spherical shape. The average elemental composition of the nanoparticles is continuously tunable via the manipulation of the spark current waveform, achieved here by varying the total resistance of the discharge circuit, that results in the variation of the [Co]/([Co] + [Au]) content from 24 to 64 at.%. The experimentally determined compositions are consistent with the results of our semiempirical spark mixing model, when using cathode fall voltages of 60 V and 28 V for Au and Co, respectively. Since the applied spark-based technique was demonstrated to be scalable even up to industrial levels, our findings may contribute to the efficient and sustainable synthesis of Au/Co nanocatalysts and their applications in the field of energy storage and in hydrogen-based fuel cells, in particular.

## Data Availability

The datasets used and/or analysed during the current study available from the corresponding author on reasonable request.
